# CRISPR/CasRx Proof-of-Concept for RNA Degradation: A Future Tool against RNA Viruses?

**DOI:** 10.3390/ph15010032

**Published:** 2021-12-27

**Authors:** Diana Perez-SanJose, Miguel Angel de la Fuente, Julia Serna Pérez, Maria Simarro, José María Eiros Bouza, Ivan Sanz-Muñoz

**Affiliations:** 1 National Influenza Center of Valladolid, Hospital Clínico Universitario de Valladolid, University of Valladolid, 47010 Valladolid, Spain; jmeirosbouza@gmail.com (J.M.E.B.); isanzm@saludcastillayleon.es (I.S.-M.); 2 Targeted Gene Modification Laboratory, Unidad de Excelencia Instituto de Biología y Genética Molecular (IBGM), 47003 Valladolid, Spain; mafuente@ibgm.uva.es (M.A.d.l.F.); juuliaserna@gmail.com (J.S.P.); msimarrogrande@gmail.com (M.S.); 3 Department of Cell Biology, Histology and Pharmacology, University of Valladolid, 47005 Valladolid, Spain; 4 Department of Biochemistry and Molecular Biology and Physiology, University of Valladolid, 47005 Valladolid, Spain; 5 Nursing Unit, Nursing Faculty, University of Valladolid, 47005 Valladolid, Spain; 6 Microbiology Service, Hospital Universitario Río Hortega, 47012 Valladolid, Spain

**Keywords:** CRISPR/Cas system, antiviral, influenza virus, CRISPR/Cas13d, CRISPR/CasRx

## Abstract

Influenza viruses provide a great threat for the human population, causing highly contagious respiratory infections that can lead to serious clinical complications. There are a limited variety of influenza antivirals, and these antivirals are subjected to the constant emergence of resistances. Therefore, the development of new antiviral strategies to combat influenza viruses and other RNA viruses must be promoted. In this work, we design a proof-of-concept of a recently described CRISPR/Cas tool that has been proposed as a possible future RNA virus antiviral, named CRISPR/CasRx. For this, we verified the efficiency of the CasRx endonuclease in the degradation of the eGFP mRNA reporter gene and we established the best conditions for, and the efficient performance of, the CRISPR/CasRx system. The results were measured by fluorescence microscopy, flow cytometry, and qRT-PCR. The analyses demonstrated a reduction in fluorescence, regardless of the amount of eGFP reporter plasmid transfected. The analyses showed an 86–90% reduction in fluorescence by flow cytometry and a 51–80% reduction in mRNA expression by qRT-PCR. Our results demonstrate that the CasRx endonuclease is an efficient tool for eGFP mRNA knockdown. Therefore, subsequent experiments could be useful for the development of a new antiviral tool.

## 1. Introduction

The presence of long stretches of tandem repeats in the genome of prokaryotes was first reported by Dr F. Mojica in 1995 [1] although its function was still unknown. These genomic regions were initially defined as short regularly spaced repeats (SRSRs) [2], and afterwards, its name was modified to clustered regularly interspaced short palindromic repeats (CRISPR) [3]. It was not until 2005 that the presence of CRISPR/Cas systems was related to their ability to confer specific immunity against foreign genetic materials, such as from bacteriophages or conjugative plasmids [4]. Thanks to different metagenomics studies on prokaryotic cells, it is currently known that the CRISPR/Cas systems are made up of arrays of palindromic repeats and CRISPR-associated proteins (Cas). These proteins are the main constituents of the CRISPR/Cas systems.

As a rudimentary kind of immune system, the Cas endonuclease is capable of recognising specific DNA sequences thanks to the complementary activity of two fragments of RNA, called CRISPR RNA (crRNA) and trans-acting CRISPR RNA (tracrRNA) [5]. However, new discoveries by Dr E Charpentier and Dr J Doudna have shown that these two guide RNA sequences can be conjugated in vitro into a single sequence, named single-guide RNA or guide RNA (sgRNA, gRNA). This in vitro gRNA is enough to produce double-stranded breaks in the target DNA, and simplifies the method for its use in vitro [6]. These discoveries caused a breakthrough in the development of gene modification techniques, and numerous research groups have since focused on the development of the CRISPR/Cas system as a tool for gene modification directed against DNA.

Despite the wide use of CRISPR/Cas systems until now, the main limitation of this system is the lack of knowledge about how exactly it can degrade the RNA. In 2018, a new subtype of the CRISPR/Cas system was described as a class 2 subtype VI-D CRISPR/Cas system, which uses a Cas13d endonuclease [7]. This Cas13d endonuclease employs a crRNA of a 22 nt spacer sequence which is customisable and allows Cas13d to deplete specific mRNA transcripts. Additionally in 2018, a group of researchers performed a computational search to identify previously undetected or uncharacterised RNA-targeting CRISPR/Cas systems, using all the prokaryotic genomes present in the database from NCBI WGS [8]. Through these analyses they were able to identify a new Cas13d endonuclease from *Ruminococcus flavefaciens* (Cas endonuclease XPD3002 or CasRx). This new system was named CRISPR/CasRx, and exhibits high in vitro efficiency and specificity to knockdown diverse RNA transcripts, as recent research has shown [9,10,11,12,13,14]. In addition, it is also one of the most compact Cas effector enzymes, as it can be easily packaged into viral vectors, among other systems [8].

In 2019, the RNA-targeting CRISPR/Cas systems were proposed for the first time as a possible antiviral strategy to combat human RNA viruses, coming up with different RNA-targeting Cas proteins [15]. Later on, due to the great crisis caused by the SARS-CoV-2 pandemic, the use of the CRISPR/CasRx system was tested for antiviral purposes by Abbott et al. [16]. They developed a prophylactic antiviral CRISPR tool in a human cell culture (human lung epithelial cells) and named it PAC-MAN. They tested this tool using synthesised genomic fragments of SARS-CoV-2, as well as using live H1N1 influenza A virus strains for infection in the cell culture. When PAC-MAN was transfected to a cell culture, this tool was able to cleave SARS-CoV-2 fragments and reduce the amount of influenza A virus infection in the cell culture. Recently, a new study was published in which a bioinformatic tool was developed alongside an online resource tool that facilitated the use of the CRISPR/CasRx to knock down RNA viruses. This tool will allow the prediction of the efficiency and specificity of virus-targeting crRNAs [17].

Although these recent studies have identified the CRISPR/CasRx system as an optimal strategy for specific RNA knockdown, there are several limitations. The study presented by Abbott et al. [16] focused on SARS-CoV-2 knockdown using the CRISPR/CasRx tool, but only used a live influenza virus because of its easy handling due to its low-level biosafety requirements. Therefore, several studies should be conducted to verify the use of this tool for influenza infections. Given the future applicability of the technique as an antiviral therapy against respiratory RNA viruses, we decided to study its efficacy under conditions as close as possible to an in vivo approach. In this regard, one important limitation of the study of Abbott et al. [16] is that it explores the efficacy of the CRISPR/CasRx system in a cell line that has been modified for the stable expression of the CasRx endonuclease. This situation will not occur in an in vivo antiviral therapy. Thus, the experimental setup is not fully adequate to explore the efficacy of the system as an antiviral therapy for influenza infections. For this reason, their experiments need to be complemented with studies that explore the safety of CasRx endonuclease in cells, including studying its off-target effects at the RNA level. Furthermore, complementary studies are needed to find the best and most efficient method for the delivery of CRISPR/CasRx in the human respiratory tract, to verify its usefulness for in vivo therapies against respiratory viruses.

Influenza is a highly contagious respiratory virus that has been circulating among humans for centuries, and yet, this disease continues to have a great global impact each year. During each epidemic season, 1000 million people are estimated to be affected worldwide, three to five million people are hospitalised each year, and an average of 650,000 people die each year due to high-severity influenza infections [18]. Due to its antigenic drift, we suffer influenza epidemics each year, and through antigenic shift, influenza viruses are able to spontaneously transfer from animal hosts to humans, with the consequent emergence of a potentially pandemic virus [19]. Although an influenza vaccine exists, its effectiveness is sub-optimal, so in cases of severe infection and illness, the use of antivirals is necessary [20]. There are limited varieties of flu antivirals and, in addition, these antivirals are subject to the constant appearance of resistances, and therefore the use of antivirals is constantly limited [21].

Taking this into account, the development of a new antiviral strategy is necessary to combat influenza virus symptoms and their severities. Optimally, this new antiviral treatment should allow a rapid industrial production and be easily adaptable to the new viral variants that emerge every year. Thus, the objective of this study is to adapt the CRISPR/CasRx technology, originally designed by Konermann et al. in 2018 [8], for future use as an antiviral drug against influenza viruses. Through this proof-of-concept, we establish the optimal conditions for the functioning of the CRISPR/CasRx system, using the eGFP (enhanced GFP) reporter gene and eGFP gRNAs.

## 2. Results

**Figure 1:**
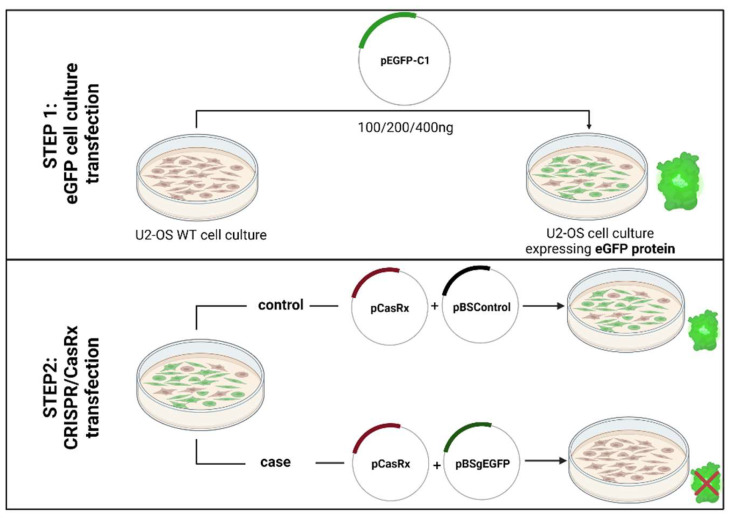
Schematic representation of the transfection protocol. Created with Biorender.com (accessed on 24 August 2021).

In this study, our main objective was the generation of a novel fluorescent tool for monitoring the efficiency of the CRISPR/CasRx as a proof-of-concept of previous research. To do this, we have transfected the plasmids for the CRISPR/CasRx system (pCasRx, pEGFP-C1, pBSgEGFP) into the U-2 OS cell line, and analysed the results by fluorescence microscopy, flow cytometry, and qRT-PCR. A representation of the protocol is shown in the Figure below (Figure 1).

### 2.1. Evaluation of CRISPR/CasRx Endonuclease Activity by Fluorescence Microscopy

**Figure 2:**
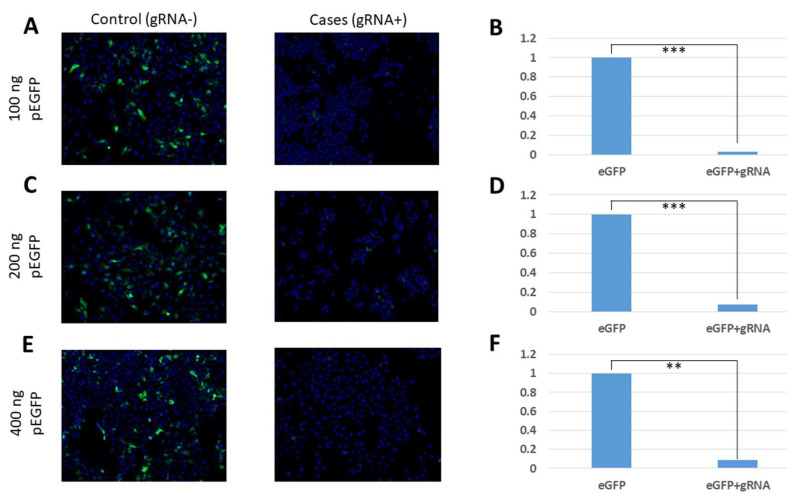
Representative fluorescence microscopy images of the cultures with gRNA (case situations) and without gRNA (control situations) (10X). From top to bottom—each row represents the different concentrations of the transfected plasmid pEGFP-C1: (**A**) 100 ng; (**C**) 200 ng; and (**E**) 400 ng. From left to right—each column represents the different situations for each condition. The first column represents the control situation without gRNA transfection and the second column represents the cell line transfected with gRNA. Each image represents a combination of two images taken at an excitation of 489 nm to allow the visualisation of eGFP fluorescence, and at 350 nm to allow the visualisation of the Hoechst dye. Exposures of 100 ms for green fluorescence and 10 ms for blue fluorescence were used. The statistical analyses of the normalised mean eGFP fluorescence in control and case situations for each pEGFP-C1 concentration: (**B**) 100 ng; (**D**) 200 ng; and (**F**) 400 ng. A two-sided *t* test with unequal variance and significance was used, indicated as: ** *p* ≤ 0.01, and *** *p* ≤ 0.001.

The analysis carried out by fluorescence microscopy for visualising the fluorescence emitted by eGFP in the transfected cell cultures showed a significant decrease in green fluorescence between the case situation (with gRNA) and the control situation (without gRNA, no CasRx activity) in all the analysed concentrations of the plasmid pEGFP-C1 (Figure 2). In all the conditions in which gRNA was transfected, a significant reduction of the florescence was observed between the control and case situations (Student-T; 100 ng *p* ≤ 0.001; 200 ng *p* ≤ 0.001; 400 ng *p* ≤ 0.01). When carrying out these experiments, it was observed that the amount of the plasmid pEGFP-C1 transfected in the cell culture did not influence the observed fluorescence.

### 2.2. Flow Cytometry Analysis of the Decrease of eGFP Fluorescence

A previous evaluation of the transfection efficiency of the plasmid pEGFP-C1 was made by means of the fluorescence-intensity study carried out by flow cytometry. For this, the different plasmids were transfected following the control and case conditions described in the materials and methods section. A sample of untransfected U-2 OS WT (wild type) cells was also included as a negative eGFP control. The transfection efficiency varied between 16.8% (condition 100 ng control) and 33.2% (condition 400 ng control). 

**Figure 3:**
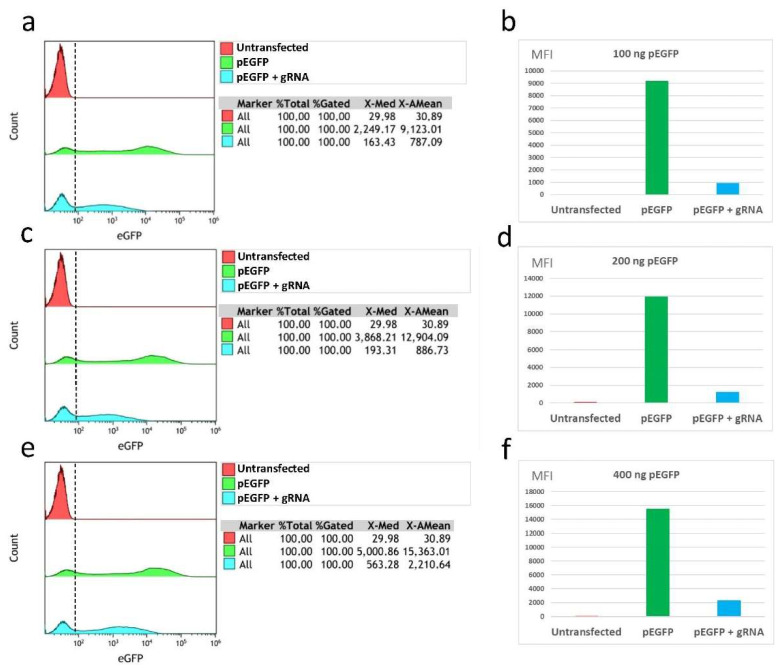
Histograms for total cell populations of each condition: (**a**) condition 100; (**c**) condition 200; (**e**) condition 400. The mean of the MFI triplicated values represented for each condition: (**b**) condition 100; (**d**) condition 200; and (**f**) condition 400. For each condition, a graph with three histograms is represented. Each histogram represents two different cell populations: the population to the left of the discontinued line corresponds to cells without fluorescence, and the rest of the population corresponds to cells that presented eGFP fluorescence. From top to bottom: untransfected cells (red); control situation transfected cells (green); and case situation transfected cells (blue) are represented in each histogram (identified in the upper box of each graph). The mean fluorescence intensity (MFI) was calculated using the arithmetic mean of eGFP fluorescence and represented using the mean of the values obtained in each triplicate. The graphs in this image are downloaded from Kaluza software.

The analysis of eGFP fluorescence reduction by flow cytometry showed that the mean fluorescence intensity (MFI) for the three triplicates was reduced in all case conditions with respect to their corresponding control conditions. A reduction of 89.1% (CI95%; 84.3–91.6) (8245.5 MFI units) for 100 ng conditions was observed, as well as a reduction of 89.8% (CI95%; 87.1–93.1) (10,687.2 MFI units) for 200 ng conditions, and a reduction of 85.8% (CI95%; 84.4–87.4) (13,247.23 MFI units) for 400 ng conditions (Figure 3).

### 2.3. Relative Expression Analysis of eGFP mRNA by qRT-PCR

**Figure 4:**
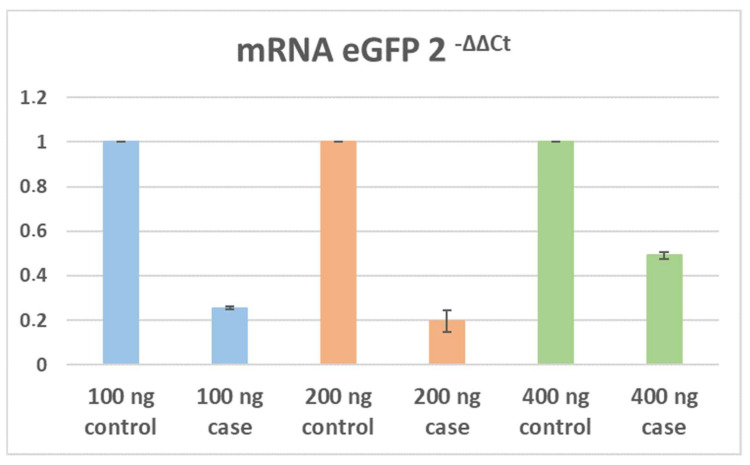
Normalised data for the relative expression of eGFP mRNA obtained by qRT-PCR; representative data for *n* = 3. The values for the transfection of the 100 ng case condition compared to the 100 ng control condition are represented in blue; the values for the transfection of the 200 ng case condition compared to the 200 ng control condition are represented in orange; and the values for the transfection of the 400 ng case condition compared to the 400 ng control condition are represented in green. The standard media error is also shown.

Using the qRT-PCR technique, it was possible to analyse the decrease in the number of transcripts of eGFP after the use of CRISPR/CasRx. This analysis was carried out under 100 ng, 200 ng, and 400 ng conditions for three biological triplicates. U-2 OS WT cells served as a negative control for eGFP expression. The qRT-PCR analysis showed a 74.4% (CI95; 73.0–75.3) decrease in the expression of the mRNA eGFP in the 100 ng case condition with respect to the 100 ng control condition, a decrease of 80.3% (CI95; 72.3–89.3) in the 200 ng case condition with respect to the 200 ng control condition, and a decrease of 50.9% (CI95; 48.9–53.7) in the 400 ng case condition with respect to the 400 ng control condition (Figure 4).

## 3. Discussion

Among the various known CRISPR-Cas type VI systems that recognise single-stranded RNA strands, in this study we have evaluated a recently described CRISPR class VI-D system (CRISPR/Cas13d) called CRISPR/CasRx [8], which could have future applications in the design of antiviral tools against human RNA viruses such as influenza [16]. This system uses the CasRx endonuclease, which has been proven to be the most effective endonuclease for RNA editing so far [8,9,10,11,12,16,22]. Some studies have achieved efficiencies of more than 90% in the destruction of RNA with the CasRx endonuclease [8].

CasRx is also the smallest effector endonuclease of CRISPR/Cas systems currently known [8] (967 amino acids long). This small size is interesting as it allows CasRx to be easily transported in plasmids to the cells of interest by adeno-associated viruses (AAV) [23,24] or by lipid nanoparticles [25,26], which would enable its future use as an antiviral system through an inhaled dosage. In future experiments, we will choose the most efficient transfection method. We have built and tested an AAV plasmid with the integrated CasRx endonuclease, thanks to which we will be able to make an “all-in-one” AAV delivery plasmid with at least one gRNA, as some papers have previously confirmed [24].

In our study, the efficiency of CasRx was verified by three different methods: fluorescence microscopy, flow cytometry, and qRT-PCR. Using our fluorescence microscopy images, we were able to verify a reduction in fluorescence in cell cultures that had been transfected with the eGFP protein expression gene, the gRNAs, and the CasRx endonuclease. In these cultures, it was possible to demonstrate the reduction of the levels of expressed eGFP proteins due to the degradation of the coding mRNA. This reduction in fluorescence was observed in all the conditions that were tested, showing that it occurs independently of the amount of transfected plasmid pEGFP-C1. On the other hand, in the control conditions, in which the plasmid carrying the specific gRNA-targeting eGFP was not transfected, fluorescence was observed despite the presence of CasRx endonuclease, which shows that this endonuclease can only be activated in the presence of gRNA.

The analyses carried out by flow cytometry and qRT-PCR showed efficacy values of CasRx comparable to those of other studies (e.g., Abbott et al. [16]). In these assays, unlike the fluorescence microscopy images, we observed that the efficacy in fluorescence reduction and eGFP gene expression varied depending on the amount of transfected plasmid pEGFP-C1. It was observed that the transfections of 100 ng and 200 ng of pEGFP-C1 obtained the best yields in fluorescence reduction through its analysis by flow cytometry (89.1% and 89.8%, respectively) and by means of analysis of the reduction of gene expression by qRT-PCR (74.4% and 80.3%, respectively). For the maximum amount of plasmid pEGFP-C1 analysed (400 ng), lower efficiencies were obtained, with fluorescence reduction values of 85.8% and a gene expression reduction of 50.9%. These results are comparable to those obtained by Abbott et al. [16], in which the authors observed that a lower amount of infecting virus in the culture was related to a greater efficiency of the endonuclease activity, obtaining greater reductions in fluorescence (78%); with greater amounts of virus they obtained smaller reductions (52%). These values can serve as a guide for future experiments in which CRISPR/CasRx is optimised for use in cell cultures with the live influenza virus.

The main differences and novelties between our work and that of Abbott et al. [16] is that we have used an AAV plasmid with multiple cloning sites (MCS) that can clone more than one gene, and this could allow the use of other transfection methods for future experiments, such as ones involving adenoviruses. On the other hand, we did not create a stable cell line that constitutively expresses the CasRx endonuclease because we transfected all the components of CRISPR/CasRx by using a Turbofect reagent. This could be closer to an in vivo scenario because CasRx is not constitutively expressed by human in vivo cells.

The main limitation of this work is that the results obtained against the eGFP mRNA reporter may not accurately represent the behaviour of the CasRx endonuclease if live viruses are used for the experiments. Therefore, it is important to optimise this tool to perform in vitro tests with different viruses to determine its efficacy in cell cultures. On the one hand, the use of liposome-mediated transfection systems limits the future use of this endonuclease in humans, so this system must be adapted to other more sophisticated and valid delivery systems for humans. For this reason, CasRx endonuclease has been cloned in an AAV plasmid, in order to use AAVs as a future transfection tool in humans. In addition, our experiments do not ensure that the eGFP mRNA has been degraded, since we only tested the reduction by qPCR. However, our experiments demonstrated a reduction in the phenotypic expression of the protein, so this ensures that the CRISPR/CasRx tool works properly. On the other hand, we have not performed cytotoxicity studies on cells once they were transfected with the CRISPR/CasRx system. Additionally, no transcriptome analysis was performed to ensure the absence of off-target effects. At the moment, we are conducting our experiments as a proof-of-concept using a gRNA against the eGFP gene. This is an in vitro situation that will not occur under any circumstances in an in vivo situation, and therefore, studies of cytotoxicity and off-targets that the CRISPR/CasRx system may cause in our cell line are not relevant. However, those experiments will be performed in the following steps of the development.

## 4. Materials and Methods

### 4.1. Study Design and Materials

A study was carried out in which a CRISPR/CasRx system, based on previous research [8], was designed for the degradation of target RNA, and through which we analysed the effectiveness in the degradation of green fluorescent protein (eGFP) mRNA in U-2 OS cells (ATCC^®^ HTB-96™, Manassas, VA, USA).

For this, three different plasmids were used. The first, called pAAV-MCS + CasRx (pCasRx), contains the CasRx protein. The gene encoding the CasRx endonuclease was amplified from plasmid pxR001 (ref #109049, Addgene, MA, USA) and cloned into pAAV-MCS (SalI and BglII restriction enzymes used) giving rise to a 7621 pb plasmid. Oligonucleotides 1 and 2 (Table 1) were used for the construction of pCasRx. The plasmid pEGFP-C1 (4731 bp) was obtained from Clontech Laboratories (Palo Alto, CA, USA). Finally, a third plasmid called pBluescript + gRNAeGFP (from now on, pBSgEGFP) was constructed, which is the plasmid that contains the gRNA against eGFP. This plasmid was constructed from an empty pBluescript (3257 bp, Agilent, CA, USA) in which a gRNA cloning backbone was introduced under the U6 promoter (oligonucleotides 5 and 6 in Table 1). The gRNA cloning backbone was obtained from plasmid pxR003 (#109053, Addgene, MA, USA). In this backbone, the 22 bp gRNA sequence of eGFP (eGFP gRNA sense: AAAC CTG CAC GCC GTA GGT CAG GGT G, eGFP gRNA antisense: AAAA CAC CCT GAC CTA CGG GGT GCA G) was cloned (BbsI restriction enzyme used), resulting in a plasmid of 3700 bp. Newly designed oligonucleotides 3 and 4 were used for the gRNA construction (Table 1).

**Figure 5:**
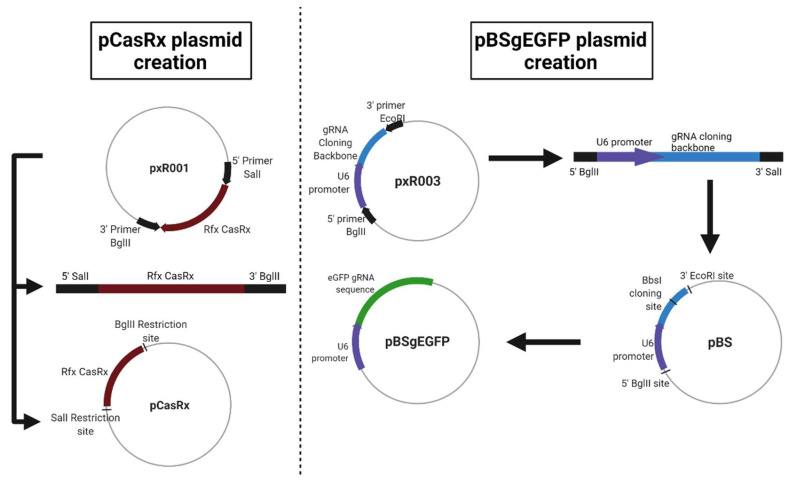
Schematic representation for the construction of each newly created plasmid. Created with Biorender.com. (accessed on 24 August 2021).

A representation of each newly constructed plasmid is shown in Figure 5.

### 4.2. Methods

To analyse the efficacy of CRISPR/CasRx in the degradation of the eGFP reporter mRNA, 80,000 cells/well were seeded into 24-well culture plates with 1 mL of complete DMEM medium and incubated at 37 °C until 90% cell confluence prior to the transfection of the plasmids. The transfection of plasmids into U-2 OS cells was performed with TurboFect (Thermo Scientific™, Waltham, MA, USA) at a 1:3 ratio (for example, 1 µg of DNA: 3 µL of Turbofect). This ratio was previously optimised in the laboratory.

Mixtures of the three plasmid DNAs (pCasRx + pEGFP-C1 + pBSgEGFP) had been prepared previously. In all cases the amount of pCasRx (400 ng), pBSgEGFP (300 ng), and OptiMEM remained constant and was used in combination with different concentrations of plasmid pEGFP-C1 (100 ng, 200 ng, or 400 ng). As a control, empty a Bluescript plasmid (pBSControl) was used. The concentration of each plasmid and conditions of transfection are shown in Table 2.

The effectiveness of CRISPR/CasRx was analysed in triplicate by three different assays: analysis of eGFP expression 24 h after transfection by flow cytometry and qRT-PCR, and at 48 h by fluorescence microscopy image analysis.

Briefly, for flow cytometry, the cells were detached using 300 µL of trypsin-EDTA. After three washes with a FACS buffer (PBS 1X, FBS 10%, Azida 0.5 gr), the pellet was analysed in the flow cytometer (Gallios Flow Cytometer). A control was added, consisting of 500,000 untransfected U-2 OS WT cells. The analysis of the flow cytometry results was performed using the Kaluza software, choosing the green fluorescence channel FL1.

In addition, an analysis was also performed by qRT-PCR 24 h after transfection. For this, an extraction of genetic material was carried out with TRIZOL and the obtained RNA was quantified using Nanodrop ND-1000 (Thermo Scientific, Waltham, MA, USA). The cDNA was synthesised by reverse transcription (RT) with random hexamers using First Strand cDNA Synthesis reagents (Thermo Scientific, Waltham, MA, USA) following the manufacturer’s recommendations. Subsequently, qPCR was performed using PowerUp SYBR Green master mix (Applied Biosystems, Waltham, MA, USA) in the LightCycler^®^ 480II thermal cycler (Roche, Basel, Switzerland). The relative expression of the genes was determined using primers that hybridise in the cDNA of the genes eGFP, human RPL18, and human GAPDH (oligonucleotides 7–12 in Table 1). The PCR program consisted of an initial denaturation process at 95 °C for ten minutes, followed by 45 cycles of 15 s at 95 °C, 30 s at 60 °C, 45 s at 72 °C, and a final extension step of five minutes at 72 °C. The GAPDH gene and RPL18 ribosomal gene were selected as constitutive genes for normalisation. The relative expression of each gene was compared with the U-2 OS WT cell line using the 2^−ΔΔCt^ method [27].

Fluorescence microscopy analysis was performed 48 h after transfection. The cells were washed three times with phosphate-buffered saline (PBS) and then fixed with 4% paraformaldehyde. Subsequently, the cell nuclei were stained with Hoescht DNA fluorescent dye (0.03%) (Hoescht 33342, Invitrogen, Waltham, MA, USA). The cells were washed again twice with PBS, and the coverslips were mounted. Fluorescence microscopy images were obtained immediately. Finally, the images taken were analysed using the ImageJ software, using the mean fluorescence values. 

### 4.3. Statistical Analysis

All the experiments were performed with three technical replications on different days. All data and statistical analyses were performed using Microsoft Excel (Microsoft Office Professional Plus 2019). For parametric data, a two-sided *t* test with unequal variance and significance was used, indicated as: * *p* ≤ 0.05; ** *p* ≤ 0.01, and *** *p* ≤ 0.001.

## 5. Conclusions

The CRISPR/Cas system that uses CasRx as an endonuclease is an effective tool for the specific removal of mRNA. The experiments presented here are the basis for the future design of antiviral tools against RNA viruses. Our results confirm that the presence of the specific gRNA against eGFP allows the activation of the CasRx endonuclease, which causes the breakdown of the target eGFP mRNA, reducing the amount of eGFP protein present in the cell. This produces a significant decrease in mRNA and the fluorescence emitted by eGFP that can be detected. In the absence of gRNA, the CasRx endonuclease is not activated, the mRNA encoding eGFP is not degraded, and the amount of eGFP protein remains high. Future work should analyse the efficacy of this tool against different live RNA viruses in cells and animals and identify compatible vehicle systems suitable for use in humans. The results of these experiments show that the CRISPR/CasRx system seems to be a useful tool for developing antivirals against RNA viruses.

## Figures and Tables

**Table 1:** Oligonucleotides designed for the construction of newly created plasmids and qRT-PCR analysis.

Denomination	Oligonucleotide Sequence
1- CasRx Sal SEN	AATGTCGACATGAGCCCCAAGAAGAAGAG
2- CasRx BglII ATS	AATAGATCTTAAGCAGCGTAATCTGGAACATC
3- gRNAeGFP BbsI SEN	AAACCTGCACGCCGTAGGTCAGGGTG
4- gRNAeGFP BbsI ATS	AAAACACCCTGACCTACGGCGTGCAG
5- U6-gRNA BglII SEN	ATTAGATCTGCGAGGGCCTATTTCCCATG
6- U6-gRNA EcoRI ATS	TAAGGAGAAAATACCGCATCAG
7- eGFP SEN	TGCAGTGCTTCAGCCGCTA
8- eGFP ATS	AGAAGATGGTGCGCTCCTG
9- RPL18 SEN	AACTGATGATGTGCGGGTTC
10- RPL18 ATS	CAGCTGGTCGAAAGTGAGG
11- GAPDH SEN	CATGACCACAGTCCATGCCATCACT
12- GAPDH ATS	TGAGGTCCACCACCCTGTTGCTGTA

The restriction enzymes used are included in the “Denomination” column. The restriction enzyme compatible ends are underlined.

**Table 2:** Composition of the plasmid DNA mixtures OptiMEM and Turbofect which were transfected per well of U-2 OS cells.

Conditions	OptiMEM	pCasRx	pEGFP-C1	pBSgEGFP	pBSControl	Turbofect
100 ng case	100 µL	400 ng	100 ng	300 ng	X	2.4 µL
100 ng control	100 µL	400 ng	100 ng	X	300 ng	2.4 µL
200 ng case	100 µL	400 ng	200 ng	300 ng	X	2.7 µL
200 ng control	100 µL	400 ng	200 ng	X	300 ng	2.7 µL
400 ng case	100 µL	400 ng	400 ng	300 ng	X	3.3 µL
400 ng control	100 µL	400 ng	400 ng	X	300 ng	3.3 µL

The “X” in the table means that plasmid was not added in the corresponding condition.

## Data Availability

Data is contained within the article.

## References

[1] Mojica F.J.M., Ferrer C., Juez G., Rodríguez-Valera F. (1995). Long stretches of short tandem repeats are present in the largest replicons of the Archaea Haloferax mediterranei and Haloferax volcanii and could be involved in replicon partitioning. Mol. Microbiol..

[2] Mojica F.J.M., Díez-Villaseñor C., Soria E., Juez G. (2000). Biological significance of a family of regularly spaced repeats in the genomes of Archaea, Bacteria and mitochondria. Mol. Microbiol..

[3] Jansen R., Van Embden J.D.A., Gaastra W., Schouls L.M. (2002). Identification of genes that are associated with DNA repeats in prokaryotes. Mol. Microbiol..

[4] Mojica F.J.M., Díez-Villaseñor C., García-Martínez J., Soria E. (2005). Intervening sequences of regularly spaced prokaryotic repeats derive from foreign genetic elements. J. Mol. Evol..

[5] Jiang F., Doudna J.A. (2017). CRISPR-Cas9 structures and mechanisms. Annu. Rev. Biophys..

[6] Jinek M., Chylinski K., Fonfara I., Hauer M., Doudna J.A., Charpentier E. (2012). A programmable dual-RNA-guided DNA endonuclease in adaptive bacterial immunity. Science.

[7] Yan W.X., Chong S., Zhang H., Makarova K.S., Koonin E.V., Cheng D.R., Scott D.A. (2018). Cas13d is a compact RNA-targeting Type VI CRISPR effector positively modulated by a WYL-domain-containing accessory protein. Mol. Cell.

[8] Konermann S., Lotfy P., Brideau N.J., Oki J., Shokhirev M.N., Hsu P.D. (2018). Transcriptome engineering with RNA-targeting Type VI-D CRISPR effectors. Cell.

[9] Mahas A., Aman R., Mahfouz M. (2019). CRISPR-Cas13d mediates robust RNA virus interference in plants. Genome Biol..

[10] Zhou H., Su J., Hu X., Zhou C., Li H., Chen Z., Xiao Q., Wang B., Wu W., Sun Y. (2020). Glia-to-neuron conversion by CRISPR-CasRx alleviates symptoms of neurological disease in mice. Cell.

[11] Sun R., Brogan D., Buchman A., Yang T., Akbari O.S. (2021). Ubiquitous and tissue-specific RNA targeting in Drosophila melanogaster using CRISPR/CasRx. J. Vis. Exp..

[12] Jiang W., Li H., Liu X., Zhang J., Zhang W., Li T., Liu L., Yu X. (2020). Precise and efficient silencing of mutant KrasG12D by CRISPR-CasRx controls pancreatic cancer progression. Theranostics.

[13] Yi W., Li J., Zhu X., Wang X., Fan L., Sun W., Liao L., Zhang J., Li X., Ye J. (2020). CRISPR-assisted detection of RNA-protein interactions in living cells. Nat. Methods.

[14] Cao Y., Zhou H., Zhou X., Li F. (2021). Conferring resistance to plant RNA viruses with the CRISPR/CasRx system. Virol. Sin..

[15] Freije C.A., Myhrvold C., Boehm C.K., Lin A.E., Welch N.L., Carter A., Metsky H.C., Luo C.Y., Abudayyeh O.O., Gootenberg J.S. (2019). Programmable inhibition and detection of RNA viruses using Cas13. Mol. Cell.

[16] Abbott T.R., Dhamdhere G., Liu Y., Lin X., Goudy L., Zeng L., Chemparathy A., Chmura S., Heaton N.S., Debs R. (2020). Development of CRISPR as an antiviral strategy to combat SARS-CoV-2 and influenza. Cell.

[17] Lin X., Liu Y., Chemparathy A., Pande T., La Russa M., Qi L.S. (2021). A comprehensive analysis and resource to use CRISPR-Cas13 for broad-spectrum targeting of RNA viruses. Cell Rep. Med..

[18] Carrat F., Flahault A. (2007). Influenza vaccine: The challenge of antigenic drift. Vaccine.

[19] Kim H., Webster R.G., Webby R.J. (2018). Influenza virus: Dealing with a drifting and shifting pathogen. Viral Immunol..

[20] Saladino R., Barontini M., Crucianelli M., Nencioni L., Sgarbanti R., Palamara A.T. (2010). Current advances in anti-influenza therapy. Curr. Med. Chem..

[21] Lampejo T. (2020). Influenza and antiviral resistance: An overview. Eur. J. Clin. Microbiol. Infect. Dis..

[22] Banerjee A., Mukherjee S., Maji B.K. (2021). Manipulation of genes could inhibit SARS-CoV-2 infection that causes COVID-19 pandemics. Exp. Biol. Med. (Maywood).

[23] Naso M.F., Tomkowicz B., Perry W.L., Strohl W.R. (2017). Adeno-associated virus (AAV) as a vector for gene therapy. BioDrugs.

[24] Nguyen T.M., Zhang Y., Pandolfi P.P. (2020). Virus against virus: A potential treatment for 2019-nCov (SARS-CoV-2) and other RNA viruses. Cell Res..

[25] Xu C.-X., Jere D., Jin H., Chang S.-H., Chung Y.-S., Shin J.-Y., Kim J.-E., Park S.-J., Lee Y.H., Chae C.-H. (2008). Poly(ester amine)-mediated, aerosol-delivered Akt1 small interfering RNA suppresses lung tumorigenesis. Am. J. Respir. Crit. Care Med..

[26] Park M.R., Han K.O., Han I.K., Cho M.H., Nah J.W., Choi Y.J., Cho C.S. (2005). Degradable polyethylenimine-alt-poly(ethylene glycol) copolymers as novel gene carriers. J. Control. Release.

[27] Livak K.J., Schmittgen T.D. (2001). Analysis of relative gene expression data using real-time quantitative PCR and the 2^−ΔΔCT^ method. Methods.

